# Prevalence and prognostic impact of chronic kidney disease and anaemia across ACC/AHA precursor and symptomatic heart failure stages

**DOI:** 10.1007/s00392-022-02027-w

**Published:** 2022-06-01

**Authors:** Louisa M. S. Gerhardt, Maren Kordsmeyer, Susanne Sehner, Gülmisal Güder, Stefan Störk, Frank Edelmann, Rolf Wachter, Sabine Pankuweit, Christiane Prettin, Georg Ertl, Christoph Wanner, Christiane E. Angermann

**Affiliations:** 1 Department of Stem Cell Biology and Regenerative Medicine, Eli and Edythe Broad Center for Regenerative Medicine and Stem Cell Research, Keck School of Medicine of the University of Southern California, Los Angeles, CA USA; 2 Comprehensive Heart Failure Centre, University and University Hospital Würzburg, Am Schwarzenberg 15, 97078 Würzburg, Germany; 3 Department of Medicine I, St Josefs-Hospital Wiesbaden, Wiesbaden, Germany; 4 Medical Centre Hamburg-Eppendorf, Institute of Medical Biometry and Epidemiology, Hamburg, Germany; 5 Department of Medicine I, University Hospital Würzburg, Würzburg, Germany; 6 Department of Internal Medicine, Cardiology, Charité – Campus Virchow Klinikum, Universitätsmedizin Berlin, and German Centre for Cardiovascular Research, Partner Site Berlin, Charité Universitätsmedizin Berlin, Berlin, Germany; 7 Clinic and Policlinic for Cardiology and Pneumology, University Hospital Leipzig, Leipzig, Germany; 8 Department of Cardiology, Philips-University Marburg, Marburg, Germany; 9 Clinical Trial Centre Leipzig, University of Leipzig, Leipzig, Germany; 10 Department of Medicine I (Nephrology), University Hospital Würzburg, Würzburg, Germany

**Keywords:** Anaemia, ACC/AHA classification, Chronic kidney disease, Comorbidity, Heart failure, Mortality

## Abstract

**Background:**

The importance of chronic kidney disease (CKD) and anaemia has not been comprehensively studied in asymptomatic patients at risk for heart failure (HF) versus those with symptomatic HF. We analysed the prevalence, characteristics and prognostic impact of both conditions across American College of Cardiology/American Heart Association (ACC/AHA) precursor and HF stages A–D.

**Methods and results:**

2496 participants from three non-pharmacological German Competence Network HF studies were categorized by ACC/AHA stage; stage C patients were subdivided into C1 and C2 (corresponding to NYHA classes I/II and III, respectively). Overall, patient distribution was 8.1%/35.3%/32.9% and 23.7% in ACC/AHA stages A/B/C1 and C2/D, respectively. These subgroups were stratified by the absence ( – ) or presence ( +) of CKD (estimated glomerular filtration rate [eGFR] < 60 mL/min/1.73m^2^) and anaemia (haemoglobin in women/men < 12/ < 13 g/dL). The primary outcome was all-cause mortality at 5-year follow-up. Prevalence increased across stages A/B/C1 and C2/D (CKD: 22.3%/23.6%/31.6%/54.7%; anaemia: 3.0%/7.9%/21.7%/33.2%, respectively), with concordant decreases in median eGFR and haemoglobin (all *p* < 0.001). Across all stages, hazard ratios [95% confidence intervals] for all-cause mortality were 2.1 [1.8–2.6] for CKD + , 1.7 [1.4–2.0] for anaemia, and 3.6 [2.9–4.6] for CKD + /anaemia + (all *p *< 0.001). Population attributable fractions (PAFs) for 5-year mortality related to CKD and/or anaemia were similar across stages A/B, C1 and C2/D (up to 33.4%, 30.8% and 34.7%, respectively).

**Conclusions:**

Prevalence and severity of CKD and anaemia increased across ACC/AHA stages. Both conditions were individually and additively associated with increased 5-year mortality risk, with similar PAFs in asymptomatic patients and those with symptomatic HF.

**Graphical abstract:**

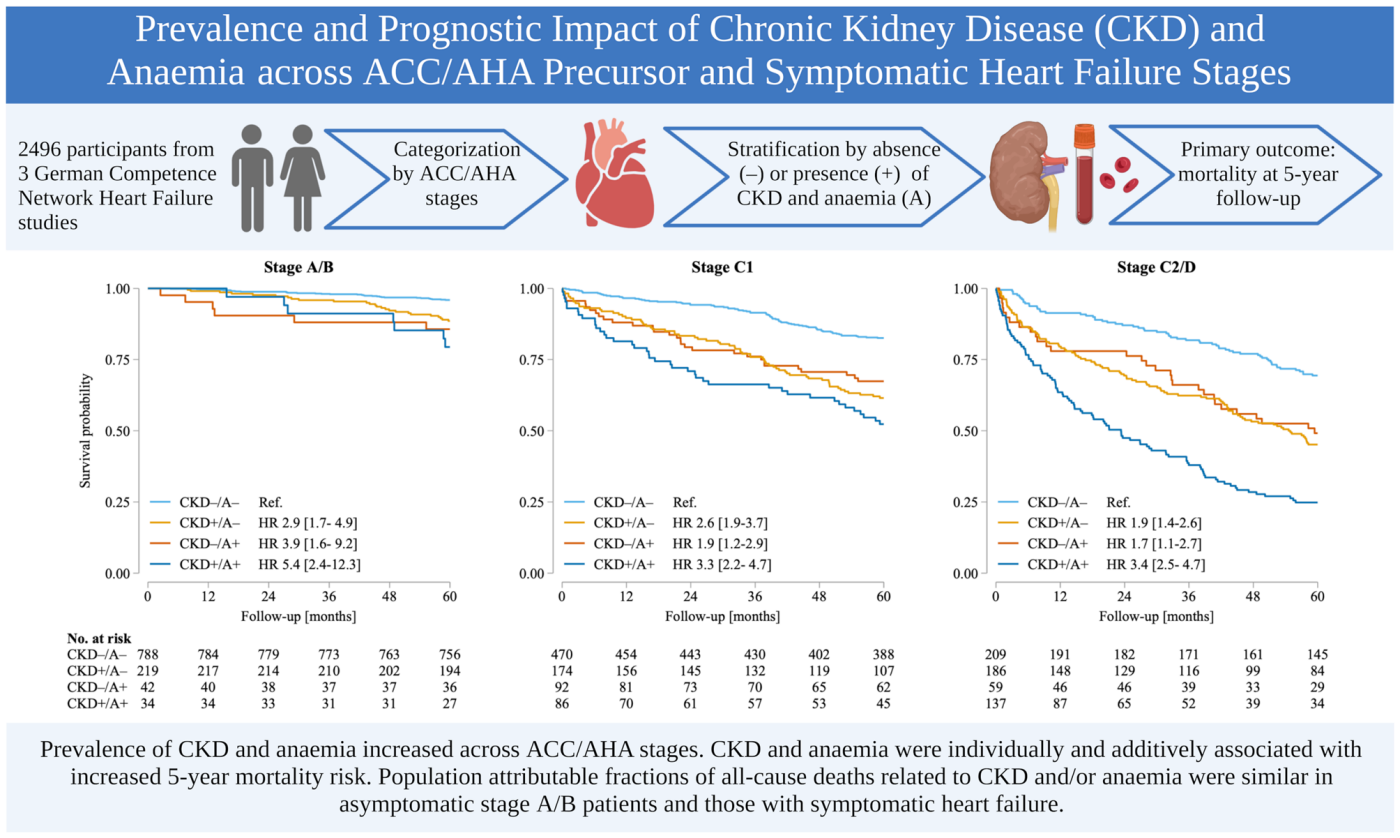

**Supplementary Information:**

The online version contains supplementary material available at 10.1007/s00392-022-02027-w.

## Introduction

Heart failure (HF) is a systemic disease involving multiple organ systems and extra-cardiac tissues [1, 2]. Chronic kidney disease (CKD) is particularly relevant because it affects up to 50% of individuals with symptomatic HF [2, 3]. Similarly, anaemia is a frequent comorbidity in HF, although prevalence rates vary, depending on the definition used and populations studied [4]. There is evidence for cardio-renal interactions across several interfaces. Bidirectional crosstalk accelerates organ dysfunction in both HF and CKD, and the development of anaemia [2, 3, 5].

CKD and anaemia have individually been extensively studied in HF populations [2–7]. Estimated glomerular filtration rate (eGFR) and haemoglobin allow quantification of disease severity. Both conditions are established risk factors that have a negative impact on clinical outcomes, with greater effects as kidney function and haemoglobin levels decline [8]. Fewer studies have investigated the relationship between the two comorbidities and the heart [9–11], and systematic analyses of their pathophysiological context, prevalence and prognostic significance across the HF trajectory are lacking.

In 2001, the ACC/AHA developed a classification that accounts for both the evolution and progression of HF [12]. It defines four stages from precursor stages A and B (termed ‘at risk for HF’ and ‘pre-HF’ in a recently proposed revised classification of HF that also incorporates this staging system [13]) through the symptomatic stages C and D [12].

Using the ACC/AHA classification enabled the study of CKD and anaemia at clearly defined stages of HF development and progression [12]. We aimed to characterise patients by ACC/AHA subgroups and determine prevalence and severity of CKD and/or anaemia, and to investigate the impact of both conditions on 5-year survival across stages.

## Methods

### Study population

Data from three non-pharmacological German Competence Network Heart Failure (CNHF) studies were used [14] (supplemental methods and Table S1). Enrolment was performed between 2004 and 2008, and 5-year follow-up between 2009 and 2014. Study participants were eligible when meeting the following criteria: high-quality echocardiogram; complete data on risk factors and comorbidities for unambiguous ACC/AHA classification; and information on survival status/date of death at 5-year follow-up. Table S1 displays total patient numbers recruited in each study versus those eligible for the current analysis. All studies were conducted according to Good Clinical Practice guidelines and Declaration of Helsinki 2002 principles, and were approved by the responsible ethics committees. All patients provided written informed consent.

### Patient assessment

The CNHF ‘basic clinical dataset’ was obtained for each participant. This includes a medical history covering symptoms, cardiac and non-cardiac comorbidities and risk factors, complete physical examination, standard laboratory assessment including NT-proBNP, 12-lead ECG and echocardiography, performed using a pre-specified imaging and reporting protocol standardised across all CNHF projects and based on American Society of Echocardiography recommendations [15]. Systolic and diastolic function, left ventricular (LV) diameter, wall thickness and wall motion abnormalities, and the presence and severity of valvular diseases were recorded where technically feasible.

Prevalence rates of CKD and anaemia were determined, and distributions of estimated glomerular filtration rate (eGFR, using the Chronic Kidney Disease Epidemiology Collaboration [CKD-EPI] equation) [16] and haemoglobin assessed from baseline serum creatinine and haemoglobin values. eGFR was not corrected for race because all patients were Caucasian. CKD was defined as eGFR < 60 mL/min/1.73m^2^, and anaemia as haemoglobin < 13.0 g/dL (males) or < 12.0 g/dL (females) (WHO criteria).

### ACC/AHA stages

Patients were assigned to ACC/AHA stages A–D using clinical, ECG, and echocardiographic criteria [12].

Stage A: No HF history, HF symptoms or echocardiographic structural or functional cardiac abnormalities, but ≥ 1 risk factor for HF development. Risk factors were: hypertension (history or blood pressure > 140/90 mmHg at enrolment); diabetes mellitus (history or antidiabetic treatment), coronary artery disease (CAD, history or medical records), and obesity (body mass index [BMI] ≥ 30 kg/m^2^).

Stage B: No current or previous HF signs and symptoms, but the presence of ≥ 1 indicator of structural heart disease, e.g., LV systolic dysfunction (LV ejection fraction [LVEF] < 50% on echocardiography); LV hypertrophy (LV mass index > 45 g/m^2.7^ [females] or > 49 g/m^2.7^ [males]) [17]; LV dilatation (LV end-diastolic diameter index [LVEDDI; LVEDD/body height [cm/m] ≥ 3.3 [females] or ≥ 3.4 [males]); LV diastolic dysfunction (early diastolic mitral flow velocity/early diastolic lengthening velocity [E/e’] > 15 or E/e’ 8.1–15 plus NT-proBNP > 220 pg/mL) [17]; presence of grade ≥ II valvular heart disease; presence of ≥ 1 regional wall motion abnormality.

Stage C: Symptomatic HF; given the wide clinical spectrum at this stage, patients were subdivided into stages C1 or C2 (corresponding to NYHA classes I/II and III, respectively), as per previously proposed symptom-based stage C sub-classification [18, 19].

Stage D: Advanced structural heart disease and severe HF signs and symptoms (NYHA class IV).

At each stage, patients were subdivided in those without CKD or anaemia (CKD–/A–), with either CKD or anaemia (CKD + /A– and CKD–/A +), or with both conditions (CKD + /A +).

### Data analysis and statistics

All data were extracted from the common CNHF database. Medical records, information from patients/relatives, and death certificates were used to ascertain vital status and date and circumstances of death in patients who died. In all studies events were adjudicated using pre-specified CNHF standard operating procedures.

Continuous variables are reported as median (quartiles), and categorical variables as absolute and relative frequencies. Comparisons across HF stages were performed using a linear trend test based on linear regression for continuous variables or logistic regression for binary variables, including an interaction term, if applicable. Two-tailed *p* < 0.05 was considered statistically significant; nominal *p* values are reported without correction for multiplicity.

The primary outcome was all-cause mortality at 5 years, and was analysed using Cox proportional hazards regression, stratified by source studies for the data to meet the proportional hazards assumption. The variables CKD, anaemia and ACC/AHA stage, plus the three-way interaction and all resulting two-way interactions were included as predictors. Stepwise backward selection based on the Wald test, starting from the three-way interaction, was employed to identify significant predictors, which remained in the model. We used three different Cox proportional hazards regression models, which differed only by the included covariates: the first included no additional covariates, the second age and sex, and the third age, sex and selected variables with prognostic relevance (leukocytes, diabetes mellitus, chronic obstructive pulmonary disease and malignancy), chosen based on existing knowledge [5, 7, 20]. For all three regression models, sensitivity analyses were performed using LVEF and NT-proBNP as surrogates for HF phenotype and disease severity.

The population attributable fraction was calculated based on ACC/AHA stage-specific Royston–Parmar models (with 4 degrees of freedom for baseline hazard function, adjusted for source studies) [21]. All analyses were performed using Stata (version 16.1).

## Results

### Patient characteristics

Of 3079 patients enrolled in the three CNHF studies, 583 were ineligible: 153 could not be categorized by the ACC/AHA classification either because quantitative echocardiographic information was lacking or information on comorbidities/risk factors was incomplete. In 430 patients, survival status or date of death at 5-year follow-up was uncertain. Among the remaining 2496 patients, 8.1%, 35.3%, 32.9%, and 23.7% were in ACC/AHA stages A, B, C1 and C2/D, respectively. Overall, patients were elderly and predominantly male (Table 1). Compared to patients with symptomatic HF, those in stages A or B were more often female and had higher blood pressure, and more than 40% of stage B patients were obese (Table 1). The prevalence of cardiac and non-cardiac comorbidities and the number of abnormal echocardiographic findings increased across ACC/AHA stages, as did levels of NT-proBNP, C-reactive protein (CRP) and leukocytes, while total cholesterol decreased. Even in the asymptomatic stages, 21% and 45% of the patients had NT-proBNP levels > 125 pg/mL (Table 1). The proportion of patients with CKD and/or anaemia was higher among patients with NT-proBNP levels > 125 pg/mL (Table S2). Across all ACC/AHA stages, patients on loop diuretics were more likely to have an NT-proBNP level > 125 pg/mL (Table S3). Average LVEF was impaired in patients with symptomatic HF, and 13% (5%) of stage C1 (C2/D) patients had preserved LVEF (Table 1).

**Table 1:** Baseline clinical and demographic characteristics of the study population by American College of Cardiology/American Heart Association heart failure stage

	ACC/AHA stage				*p*_trend_
	A (*n* = 202)	B (*n* = 881)	C1 (*n* = 822)	C2/D (*n* = 591)	
*Demographics/clinical characteristics*					
Age, years	64 (57; 69)	67 (62; 72)	66 (56; 74)	72 (60; 78)	< 0.001
Female, *n* (%)	102 (50.5)	407 (46.2)	213 (25.9)	211 (35.7)	< 0.001
Systolic blood pressure, mmHg	144 (133; 158)	150 (137; 164)	123 (110; 140)	120 (110; 130)	< 0.001
Heart rate, beats/min	72 (65; 81)	68 (61; 78)	72 (64; 80)	72 (64; 80)	0.956
Body mass index, kg/m^2^	26 (25; 29)	29 (27; 32)	27 (24; 30)	27 (24; 30)	0.911
NYHA class			2.0 (2.0; 2.0)	3.0 (3.0; 3.0)	
NYHA class, *n* (%)					
I			98 (11.9)		
II			724 (88.1)		
III				544 (92.0)	
IV				47 (8.0)	
*Comorbidities/risk factors*, *n* (%)					
Arterial hypertension*	192 (95.0)	864 (98.1)	633 (77.0)	438 (74.2)	< 0.001
Coronary artery disease	18 (8.9)	182 (20.7)	371 (45.1)	290 (49.1)	< 0.001
Obesity†	27 (13.6)	373 (42.4)	218 (26.7)	159 (27.3)	0.006
Diabetes mellitus‡	50 (24.8)	224 (25.5)	226 (27.5)	228 (38.6)	< 0.001
Chronic kidney disease§	45 (22.3)	208 (23.6)	260 (31.6)	323 (54.7)	< 0.001
Anaemia||	6 (3.0)	70 (7.9)	178 (21.7)	196 (33.2)	< 0.001
Atrial fibrillation	2 (1.0)	30 (3.4)	172 (20.9)	183 (31.0)	< 0.001
COPD^#^	9 (4.5)	55 (6.2)	106 (12.9)	117 (19.8)	< 0.001
Malignancy**	19 (9.4)	71 (8.1)	86 (10.5)	74 (12.5)	0.132
Peripheral arterial disease	6 (3.0)	45 (5.1)	74 (9.0)	76 (12.9)	< 0.001
Echocardiography					
LVEF, %	62 (57; 66)	60 (55; 65)	33 (26; 39)	30 (24; 35)	< 0.001
LVEF < 50%, *n* (%)		51 (5.8)	714 (86.9)	559 (94.6)	< 0.001
LV hypertrophy††, *n* (%)		800 (91.4)	628 (83.7)	468 (89.1)	0.157
LV dilatation, *n* (%)		92 (10.5)	458 (59.2)	391 (71.4)	< 0.001
Diastolic dysfunction, *n* (%)		204 (23.2)	165 (20.1)	106 (17.9)	0.016
Wall motion abnormalities, *n* (%)		167 (19.0)	527 (66.6)	422 (73.4)	< 0.001
Valvular disease ≥ II, *n* (%)		96 (10.9)	202 (24.6)	219 (37.1)	< 0.001
*Laboratory parameters*					
Haemoglobin, g/dL	14.2 (13.5; 15.1)	14.1 (13.3; 14.9)	14.1 (12.8; 15.1)	13.4 (12.0; 14.7)	< 0.001
eGFR, mL/min/1.73m^2^	75 (62; 85)	72 (61; 85)	71 (55; 88)	58 (40; 77)	< 0.001
Leukocytes, 10^9^/L	6.5 (5.5; 7.8)	6.5 (5.6; 7.5)	7.3 (6.2; 9.0)	8.0 (6.5; 10.0)	< 0.001
C-reactive protein, mg/L	1.2 (0.7; 2.9)	1.9 (1.0; 3.7)	5.0 (2.1; 14.0)	9.5 (5.0; 27.1)	< 0.001
Total cholesterol, mg/dL	210 (178; 234)	199 (175; 227)	180 (150; 209)	168 (139; 204)	< 0.001
NT-proBNP‡‡, pg/mL	64 (38; 108)	108 (55; 209)	1279 (342; 3667)	4298 (1396; 9979)	< 0.001
NT-proBNP > 125 pg/mL, *n* (%)‡‡	42 (20.8)	397 (45.1)	588 (89.9)	443 (98.0)	< 0.001
*Antihypertensive/heart failure medications*, *n* (%)					
ACEI and/or ARB	99 (49.3)	569 (64.9)	735 (89.4)	512 (86.8)	< 0.001
Beta-blocker	63 (31.2)	478 (54.3)	676 (82.2)	472 (79.9)	< 0.001
Mineralocorticoid receptor antagonist	2 (1.0)	11 (1.2)	299 (36.4)	278 (47.0)	< 0.001
Thiazide	76 (37.6)	385 (43.7)	209 (25.4)	132 (22.3)	< 0.001
Loop diuretic	11 (5.4)	92 (10.4)	521 (63.4)	472 (79.9)	< 0.001

Values are median (quartiles), or number of patients (%)

*ACEI *angiotensin-converting enzyme inhibitor, *ACC/AHA *American College of Cardiology/American Heart Association, *ARB *angiotensin receptor blocker, *COPD *chronic obstructive pulmonary disease, *eGFR *estimated glomerular filtration rate, *LV *left ventricular, *LVEF *left ventricular ejection fraction, *NT-proBNP* N-terminal pro b-type natriuretic peptide; NYHA, New York Heart Association

^*^Arterial hypertension was defined as systolic blood pressure ≥ 140 mmHg, diastolic blood pressure ≥ 90 mmHg and / or documented history of hypertension

^†^Obesity was defined as body mass index ≥ 30 kg/m^2^

^‡^Diabetes mellitus was defined based on antidiabetic therapy and/or history of diabetes mellitus

^§^Chronic kidney disease was defined as eGFR < 60 mL/min/1.73m^2^

||Anaemia was defined as haemoglobin < 12 g/dL in women, < 13 g/dL in men (World Health Organization criteria)

^#^COPD was defined based on anti-obstructive therapy and/or history of COPD

^**^Malignancy denotes cured and uncured malignant disease

^††^LV hypertrophy was measured in 2335 patients

^‡‡^NT-proBNP missing values at Stage C1 (20.4%) and C2/D (23.5%); all other variables were measured in > 95% of the study population

### Prevalence and severity of CKD and anaemia

**Fig. 1:**
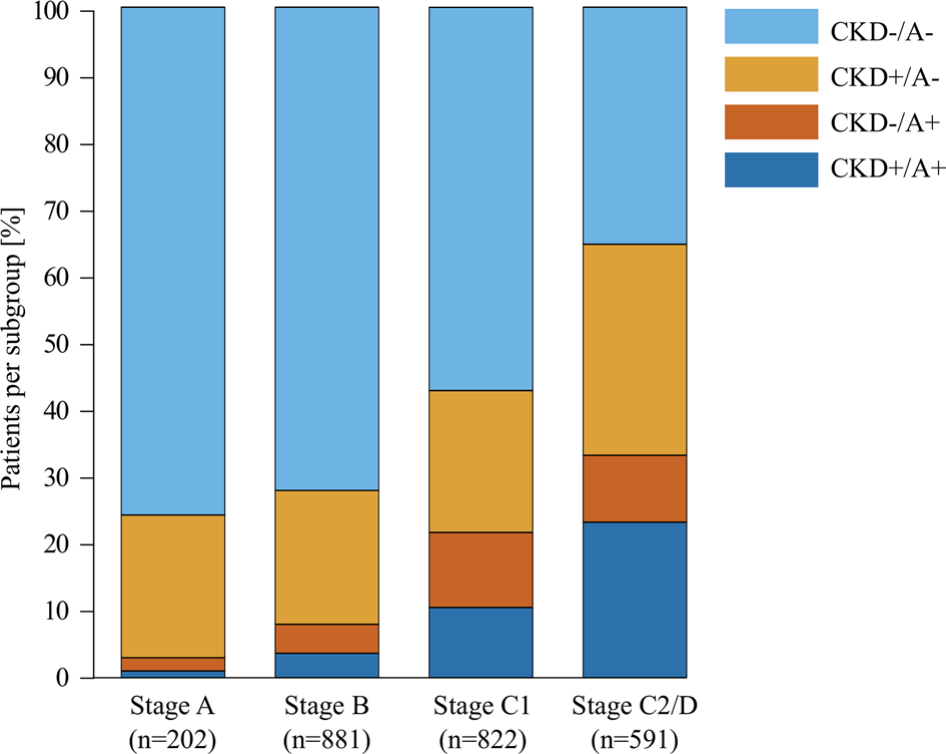
Prevalence of chronic kidney disease (CKD) and/or anaemia by American Heart Association/American College of Cardiology (ACC/AHA) stages (*n* = 2496). Stacked bar chart showing the proportion of patients with ( +) or without ( – ) CKD and/or anaemia (A) at each ACC/AHA stage

Prevalence of CKD and/or anaemia increased progressively across stages (Fig. 1). The proportion of patients with both comorbidities increased from 1.0% in stage A to 3.6%, 10.5% and 23.2% in stages B, C1 and C2/D, respectively, while the proportion with neither comorbidity declined, from 75.7% in stage A to 35.4% in stage C2/D (Fig. 1). Even at stages A or B, > 20% of the patients had CKD (Table 1), and prevalence rose with each stage (to > 50% at stages C2/D). Anaemia was uncommon in stages A and B, but prevalence increased at symptomatic stages (Table 1).

**Fig. 2:**
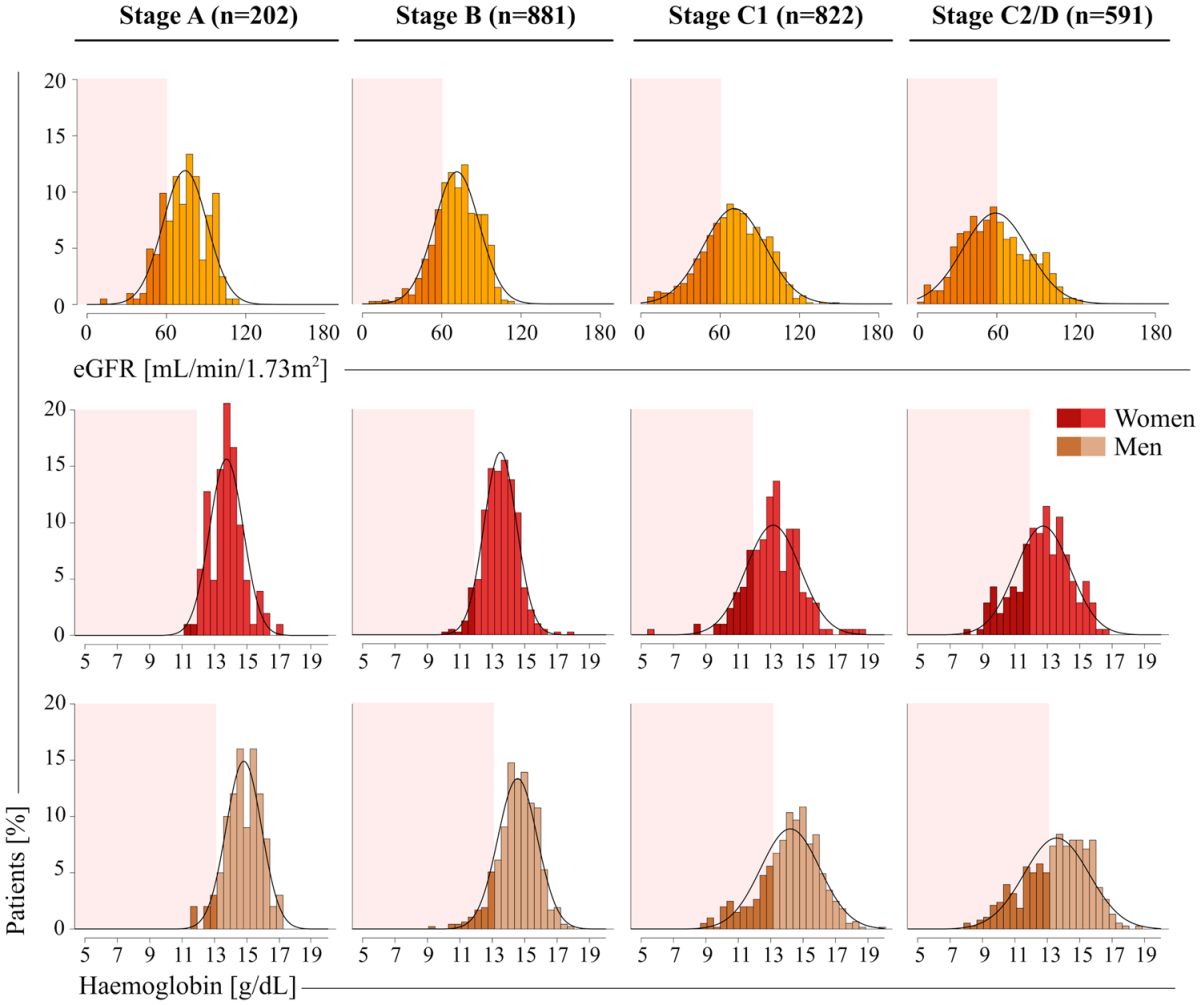
Estimated glomerular filtration rate (eGFR) and haemoglobin values by American Heart Association/American College of Cardiology (ACC/AHA) stages. Bar chart shows the distribution of eGFR and haemoglobin values by ACC/AHA stage. Darker shades of yellow indicate an eGFR < 60 mL/min/1.73m^2^ and darker shades of red/brown indicate a haemoglobin value < 12/ < 13 g/dL in females/males

Figure 2 shows distributions of eGFR and haemoglobin values at each ACC/AHA stage. In participants with CKD (but not those with a baseline eGFR ≥ 60 mL/min/1.73m^2^), median [IQR] eGFR values were lower in advanced ACC/AHA stages (54 [47–59], 52 [45–57], 47 [36–54] and 42 [32–51] mL/min/1.73m^2^ in A, B, C1, and C2/D, respectively; p < 0.001). Correspondingly, in patients with anaemia median [IQR] haemoglobin values were lower in advanced ACC/AHA stages (11.6 [11.2–11.9], 11.7 [11.5–11.8], 11.3 [10.7–11.6] and 11.1 [9.8–11.6] g/dL in women and 12.3 [11.8–12.7], 12.5 [11.7–12.8], 11.8 [10.6–12.5] and 11.6 [10.4–12.3] g/dL in men across stages A, B, C1, and C2/D, respectively; both *p* < 0.001); haemoglobin levels were similar at all stages in non-anaemic patients.

ACC/AHA stage B patients with CKD and/or anaemia were more likely to be on angiotensin-converting enzyme (ACE) inhibitors or angiotensin receptor blockers (ARBs), while stage C2/D patients with CKD and/or anaemia were less likely taking ACE inhibitors, ARBs, mineralocorticoid receptor antagonists (MRAs) and thiazide diuretics than patients without these comorbidities (Table S4). Across all stages, patients with CKD and/or anaemia received more loop diuretics (Table S4).

### Prognostic importance of CKD and anaemia

**Fig. 3:**
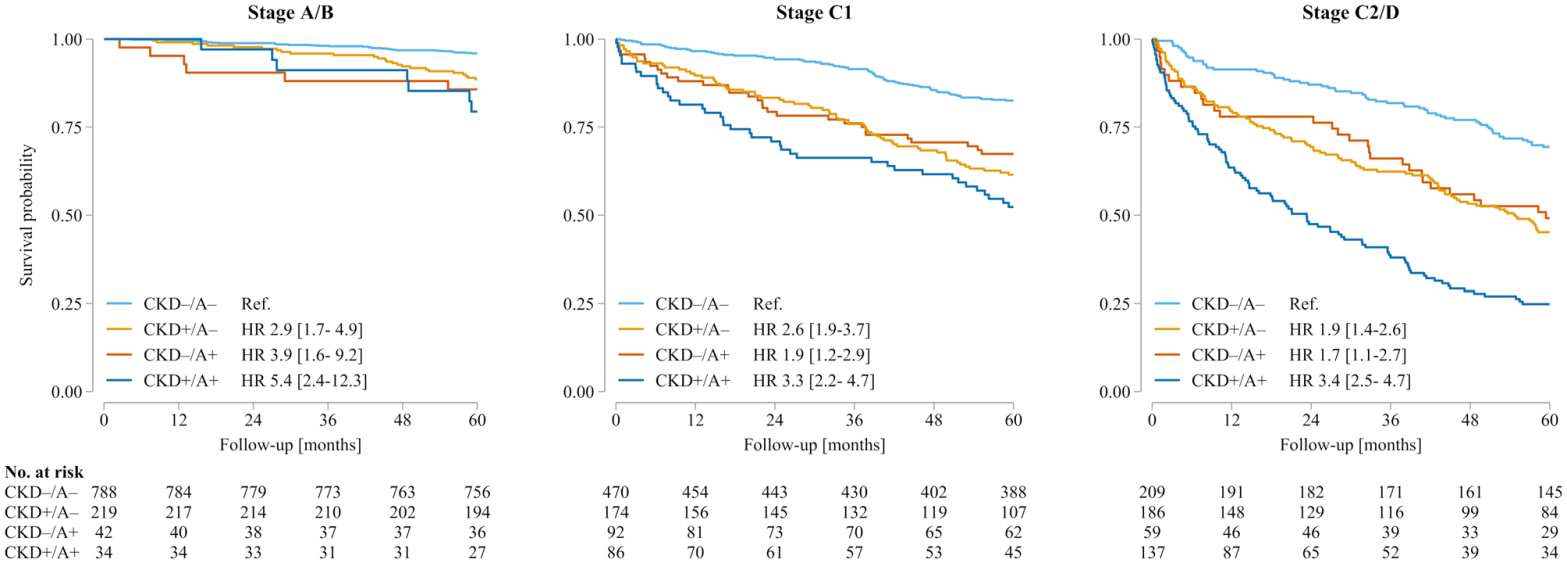
Effects of chronic kidney disease (CKD) and/or anaemia (A) on 5-year survival stratified by American Heart Association/American College of Cardiology stages (*n* = 2496). Patient numbers at risk in each subgroup according to the presence or absence of CKD and/or A are given below each panel. + , present;  – , absent; HR, hazard ratio [95% confidence interval]

In total, 589 patients (23.6%) died during follow-up; all-cause death occurred in 70 (6.5%), 220 (26.8%) and 299 (50.6%) patients with ACC/AHA stages A/B, stage C1 or stages C2/D, respectively. Mortality rates increased along the disease trajectory, and were higher in patients with CKD or anaemia. If both comorbidities were present, their adverse prognostic effects were additive (Table 2; Fig. 3). CKD and anaemia remained independent predictors of mortality risk after multivariable adjustment (Table 2), and sensitivity analyses demon

**Table 2:** Frequencies of all-cause mortality within subgroups (top panel) and multivariable adjusted models for all-cause mortality by the presence/absence of chronic kidney disease and/or anaemia

	**Frequencies of all-cause mortality within subgroups,** ***n***/***N* (%)**		
	Stage A/B	Stage C1	Stage C2/D
Total population	70/1083 (6.5)	220/822 (26.8)	299/591 (50.6)
CKD–/A–	32/788 (4.1)	82/470 (17.4)	64/209 (30.6)
CKD + /A–	25/219 (11.4)	67/174 (38.5)	102/186 (54.8)
CKD–/A +	6/42 (14.3)	30/92 (32.6)	30/59 (50.9)
CKD + /A +	7/34 (20.6)	41/86 (47.7)	103/137 (75.2)

	**Hazard ratio (95% CI);** ***p* value**		
	**Model 1 (unadjusted)**		
	Stage A/B	Stage C1	Stage C2/D
HF stages vs stage A/B	Reference	1.9 [1.2–2.9]; *p* = 0.003	3.4 [2.2–5.3]; *p* < 0.001
		All patients	
CKD+ vs CKD-		2.1 [1.8–2.6]; *p* < 0.001	
A + vs A –		1.7 [1.4–2.0]; *p* < 0.001	
CKD + /A + vs CKD – /A – *		3.6 [2.9–4.6]; *p* < 0.001	
	**Model 2 (adjusted for age and sex)**		
HF stages vs stage A/B	Reference	1.7 [1.1–2.7]; *p* = 0.010	2.9 [1.9–4.6]; *p* < 0.001
CKD + vs CKD –		1.6 [1.4–1.9]; *p* < 0.001	
A + vs A –		1.5 [1.2–1.8]; *p* < 0.001	
CKD + /A + vs CKD – /A – *		2.4 [1.9–3.1]; *p* < 0.001	
	**Model 3 (adjusted for age, sex, leukocytes, diabetes mellitus, malignancy and COPD)**		
HF stages vs stage A/B	Reference	1.6 [1.1–2.5]; *p* = 0.025	2.5 [1.6–4.0]; *p* < 0.001
CKD + vs CKD –		1.6 [1.3–1.9]; *p* < 0.001	
A + vs A –		1.5 [1.2–1.7]; *p* < 0.001	
CKD + /A + vs CKD – /A – *		2.3 [1.8–2.9]; *p* < 0.001	

*A*  anaemia, *CI *confidence interval, *CKD *chronic kidney disease, *COPD *chronic obstructive pulmonary disease, *n* number of patients with endpoint event, *N* total number of patients in subgroup,  +  = present,  –  = absent

^*^Linear combination (product) of the effects of CKD and A

strated their negative prognostic impact irrespective of disease severity and HF phenotype as assessed by NT-proBNP and LVEF (Table S5).

### Absolute mortality risk versus population attributable fraction

**Fig. 4:**
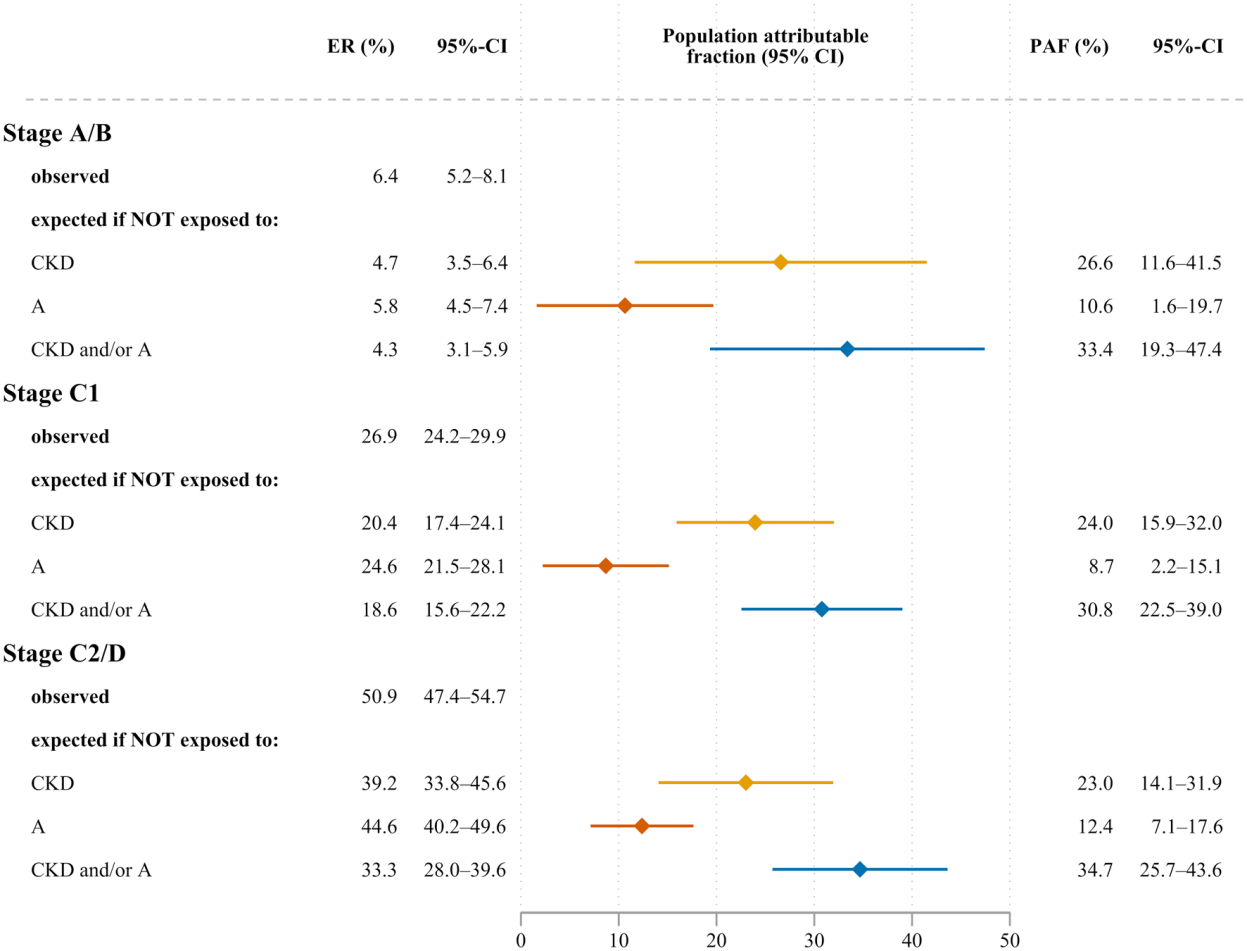
Population attributable fraction (PAF) of all-cause mortality at 5-year follow-up related to chronic kidney disease (CKD) and/or anaemia (A) by American Heart Association/American College of Cardiology stages (ACC/AHA) (*n* = 2496). PAF expresses the estimated fraction of all deaths that would not have occurred if there had been no exposure. *ER *event rate, *CI *confidence interval. CKD and/or A denotes patients with either CKD or anaemia or both conditions

Five-year survival rates for CKD–/A– patients were highest (95.9%, 82.6% and 69.4% for HF stages A/B, C1, and C2/D, respectively), while CKD + /A + patients had the lowest five-year survival rates across all ACC/AHA stages (79.4%, 52.3% and 24.8%, respectively). The population attributable fractions (PAFs) for all-cause mortality related to CKD or anaemia at 5-year follow-up did not differ substantially across ACC/AHA categories (Fig. 4). This implies that similar proportions of incident deaths across asymptomatic and symptomatic stages were related to CKD and/or anaemia. Given the low absolute mortality risk at precursor stages, this concerned only a few stage A/B patients, whereas the actual number of deaths attributable to CKD and/or anaemia was much higher at ACC/AHA stages C1 and C2/D (Fig. 4).

### Patient characteristics associated with mortality risk

Across all ACC/AHA stages, patients who died were older and had a higher disease burden (Table S6). Not only eGFR and haemoglobin values, but also BMI, proportion of obese patients and total cholesterol levels were lower, while NT-proBNP levels, inflammation markers and the percentage of patients on loop diuretics were higher than in survivors (Table S6). At stage C2/D, patients who died were less likely to receive ACE inhibitors/ARBs, beta-blockers and MRAs (Table S6).

### Causes of death

The cause of death was known for 79% of the study population. At stages A/B, the most common cause of death was non-cardiovascular (50% of patients), whereas the majority of patients with symptomatic HF (stages C1 and C2/D) died from cardiovascular causes, most often HF or arrhythmias (Table S7).

## Discussion

This study used the AHA/ACC classification [12] to triage a large sample including asymptomatic patients at risk for HF or with pre-HF, and patients with symptomatic HF, into subgroups with disease stages A-D. All participants were uniformly characterised, and the prevalence and prognostic impact of CKD and anaemia was examined across stages. We report three novel findings: (1) CKD, but not anaemia, is frequent at asymptomatic precursor stages; (2) both prevalence and severity of CKD and anaemia rise with disease severity; and (3), CKD and/or anaemia modulate 5-year mortality risk individually and additively in a similar fashion across the entire ACC/AHA spectrum.

### Disease evolution

Our data demonstrate increasing multiorgan involvement along stages A–D. Given the definition of the precursor stages, high prevalence rates of obesity, hypertension and diabetes mellitus were less surprising than an eGFR reduction to < 60 mL/min/1.73m^2^ in more than 20% of stage A/B patients. The kidney is a target site for hypertensive end-organ damage [22, 23]. Many stage A/B patients were obese and had insufficiently controlled hypertension, which likely contributed to early CKD development. In contrast, anaemia was less common in asymptomatic patients; its prevalence and severity rose in parallel with that of other non-cardiac and cardiovascular disorders as patients became symptomatic. The extent to which CKD and anaemia acted and interacted as disease mediators in our patients, or whether these conditions were just markers of the complex systemic processes implicated in HF development and progression, cannot be clarified from our observational data.

### Cardio-renal anaemia syndrome (CRAS)

In the course of HF development, even mild dysfunction of non-cardiac organs may trigger clinical manifestations [24, 25]. The interplay between CKD, anaemia and clinically overt HF has been described as CRAS, reflecting adverse effects of these conditions on HF progression and vice versa [23, 24].

Evidence from the literature suggests that even minor abnormalities in kidney function can impair its capacity to regulate extracellular fluid volume [24–26]. Volume expansion augments preload and increases oxygen demand, which can lead to LV hypertrophy and dilation, and thus accelerate progression to overt HF [24, 26]. Progressive kidney dysfunction enhances inflammation and activation of the sympathetic nervous system and the renin–angiotensin–aldosterone system [2, 3, 5]. Concurrently, CKD and HF can contribute to anaemia development and progression via haemodilution, bone marrow suppression and reduced endogenous erythropoietin production [4, 6, 24]. Inflammation may also worsen anaemia by promoting iron deficiency [27]. When haemoglobin levels decrease, oxygen carrying capacity declines, which can further add to cardiac workload by increasing heart rate and stroke volume, and promote myocardial remodelling, thus completing the vicious cycle of CRAS [2, 6, 24, 26].

Consistent with this pathophysiological cascade we found—among other systemic changes—increasingly abnormal distributions of eGFR and haemoglobin values, higher leukocyte counts and serum CRP levels, and a > 20-fold rise in the percentage of CKD + /A + patients across the ACC/AHA stages, which highlights the syndromal character of the disease [2, 4, 5]. Remarkably, significant proportions of asymptomatic patients had NT-proBNP levels above 125 pg/mL, which supports the concept that this biomarker may be elevated before HF symptoms and signs develop [28]. Levels were higher in patients with CKD and/or anaemia, and among those on loop diuretics, higher proportions of stage A and B patients had elevated NT-proBNP levels. Our data illustrate also that even these small elevations are associated with higher 5-year mortality risk in stage A/B patients.

Previously, Lam et al. observed that both renal and haematopoietic system dysfunction had similar hazard ratios for incident HF in participants of the Framingham Heart Study [25]. The current findings add to these data, showing that while absolute 5-year mortality risk rises steeply throughout the ACC/AHA spectrum, CKD and anaemia individually and additively increased patients’ mortality risk across stages A/B, C1 and C2/D with similar proportions of incident deaths related to CKD and/or anaemia.

### Prognostic implications

Despite the lower absolute mortality risk in asymptomatic patients, the similar PAFs of mortality risk related to CKD and/or anaemia in stage A/B patients compared with symptomatic patients call for vigorous preventive efforts and early adequate treatment of risk factors/comorbidities such as hypertension, obesity, and diabetes mellitus. Although the number needed to treat is larger, the potential lifetime yield for the many stage A/B patients with preventable risk factors, in whom end-organ damage could be attenuated or averted, might be much greater than that achievable in populations with more advanced HF [29].

Previous studies have also highlighted the combined impact of CKD and anaemia on mortality risk in symptomatic HF. For example, a large observational study based on Medicare data reported annual mortality rates of 13% in HF patients without CKD or anaemia and 23% in those with both comorbidities [9]. During 2.5-years’ follow-up, Lu et al. found a 51% mortality rate in CKD + /A + HF patients versus 26% in HF patients without these comorbidities [10]. Both studies also identified an additive effect of CKD and anaemia on mortality risk [9, 10]. Similar effects of anaemia and CKD were reported by Scrutinio et al. for the composite of death or urgent heart transplant [11], consistent with our observation that concurrent CKD and anaemia doubled mortality risk also in stage C2/D HF patients, of whom < 25% survived 5 years. Iorio et al. reported PAFs of 22% and 21%, respectively, for CKD and anaemia in a community-based chronic HF population irrespective of HF phenotype, but did not specifically consider the combined risks of both [7].

Our results demonstrate that although causalities seem more complex at symptomatic HF stages, CKD and anaemia retained their prognostic significance even after multivariable adjustment. Stage C2/D patients with these condition were also less often on ACE inhibitors/ARBs and MRA, which illustrates that CRAS may be a barrier to the use of life-saving therapies at advanced HF stages [23].

The current data also showed higher prevalence rates of CKD, anaemia and other non-cardiac and cardiac comorbidities in patients who died compared with survivors. Recent evidence suggests that HF populations may benefit if non-cardiac comorbidities are treated [1]. In particular, intravenous iron replacement therapy has been shown to improve rehospitalisation rates and quality of life in HF patients with iron deficiency [30, 31]. Regular screening for this common comorbidity, which often accompanies anaemia [4, 32], is recommended by the most recent European Society of Cardiology guidelines for the diagnosis and treatment of acute and chronic HF [1]. Interestingly, a secondary analysis from one trial showed that iron replacement therapy was also associated with eGFR improvements [33]. While laboratory screening for iron deficiency was not widely recommended at the time our data were collected, all HF patients diagnosed with anaemia today should have their iron status assessed [1], although reduction of the high mortality risk of iron-deficient HF patients by intravenous iron replacement has yet to be demonstrated [30].

## Limitations

Several limitations must be mentioned. Age and sex distribution was uneven across subgroups, and symptomatic patients with preserved LVEF were underrepresented because two of the three CNHF studies, from which participants were enrolled, included only patients with reduced LVEF. This might have impacted our results, given that in some previous studies the relative contribution of non-cardiac versus cardiac disease burden to mortality risk differed in HF patients with preserved versus reduced LVEF [5], although others have not confirmed this finding [7]. Sensitivity analyses with adjustment for demographic factors, NT-proBNP and LVEF consolidate our findings, but residual confounding or effect modification by other factors may have occurred. In particular, risk estimates might have been affected in a non-linear fashion by conditions such as hypertension or hypercholesterolemia that increase cardiovascular risk at early disease stages, but have been associated with the opposite impact in symptomatic HF patients [19].

Patient phenotyping was performed only once, which precludes description of time-dependent changes within each ACC/AHA stage, and additional information on possible causes of anaemia (e.g., biomarkers of iron deficiency) and CKD (e.g., albumin-creatinine ratio) was not available from the CNHF basic clinical data set. Due to its high prevalence and prognostic significance in HF patients [1, 32], the latest European Society of Cardiology guidelines for the diagnosis and treatment of acute and chronic HF recommend screening for iron deficiency [1], which should be treated appropriately [30]. Not all medications currently recommended for the prevention and treatment of HF and CKD were available at the time of study enrolment and follow-up (e.g., angiotensin receptor–neprilysin inhibitors and sodium-glucose co-transporter 2 inhibitors) [1]. Due to small numbers in patient subgroups, stratification according to the presence and absence of CKD and anaemia was binary and the prognostic impact of different degrees of severity of both CKD and anaemia across ACC/AHA stages was not assessed. Retrospective assignment of ACC/AHA stages could have biased classification, although survival data were collected prospectively, and staging was performed without knowledge of survival status. Due to smaller numbers of stage A and D patients, individual prognostic assessment was not possible in these subgroups, although characteristics of patients at stages A and B could be described separately. Another important limitation of our study is lack of generalisability. Patients were recruited in one country (Germany), and all were Caucasian. For these reasons, future validation of our results in other cohorts/ethnicities undergoing contemporary pharmacotherapies is warranted.

## Conclusions

Results of this observational study demonstrated that the prevalence and severity of CKD and anaemia increase across ACC/AHA stages A–D. The findings support the concept that kidney dysfunction is an early trigger of HF development, while the prevalence of anaemia rises as HF becomes symptomatic. Both conditions were individually and additively associated with increased 5-year mortality risk. While absolute mortality risk increased across ACC/AHA stages, the PAFs of all-cause deaths related to CKD and/or anaemia were similar in stage A/B patients and those with symptomatic HF. Our findings highlight an important role for CKD and anaemia during HF development and progression, and support the implementation of stringent preventive and therapeutic strategies starting at asymptomatic stages.

## Supplementary Information

Below is the link to the electronic supplementary material.

Supplementary file1 (DOCX 72 KB)
