# Supplementary material for: Prevalence and prognostic impact of chronic kidney disease and anaemia across ACC/AHA precursor and symptomatic heart failure stages
Source: Clin Res Cardiol. 2022 Jun 1;112(7):868–79. doi: 10.1007/s00392-022-02027-w (PMC10293329; doi:10.1007/s00392-022-02027-w)
Supplement: Supplementary file 1 — Supplementary file1 (DOCX 72 KB) [file 392_2022_2027_MOESM1_ESM.docx]

**SUPPLEMENTAL MATERIAL**

**Prevalence and Prognostic Impact of Chronic Kidney Disease and Anaemia across ACC/AHA Precursor and Symptomatic Heart Failure Stages**

Louisa M.S. Gerhardt MD^1#^, Maren Kordsmeyer MD^2,3#^, Susanne Sehner MSc^4^, Gülmisal Güder MD, PhD^2,5^, Stefan Störk MD, PhD^2,5^, Frank Edelmann MD^6^, Rolf Wachter MD^7^, Sabine Pankuweit PhD^8^, Christiane Prettin PhD^9^, Georg Ertl MD^2^, Christoph Wanner MD^2,10^, Christiane E. Angermann MD^2*^

^#^These authors contributed equally

^1^Department of Stem Cell Biology and Regenerative Medicine, Eli and Edythe Broad Center for Regenerative Medicine and Stem Cell Research, Keck School of Medicine of the University of Southern California, Los Angeles, CA, USA

^2^Comprehensive Heart Failure Centre, University and University Hospital Würzburg, Würzburg, Germany

^3^St Josefs-Hospital Wiesbaden, Department of Medicine I, Wiesbaden, Germany

^4^Medical Centre Hamburg-Eppendorf, Institute of Medical Biometry and Epidemiology, Hamburg, Germany

^5^Department of Medicine I, University Hospital Würzburg, Würzburg, Germany

^6^Department of Internal Medicine, Cardiology, Charité – Campus Virchow Klinikum, Universitätsmedizin Berlin, and German Centre for Cardiovascular Research, partner site Berlin, Charité Universitätsmedizin Berlin, Berlin, Germany

^7^Clinic and Policlinic for Cardiology and Pneumology, University Hospital Leipzig, Leipzig, Germany

^8^Department of Cardiology, Philips-University Marburg, Marburg, Germany

^9^Clinical Trial Centre Leipzig, University of Leipzig, Leipzig, Germany

^10^Department of Medicine I (Nephrology), University Hospital Würzburg, Würzburg, Germany

**SUPPLEMENTAL METHODS**

**Non-pharmacological Competence Network Heart Failure studies contributing patients**

Eligible patients were participants from three different studies (**Table S1**). Two were prospective cohort studies (Inflammatory/Familial Dilated Cardiomyopathy: Is There a Link to Autoimmune Diseases? [IKARIUS] [1] and Diagnostic Trial on Prevalence and Clinical Course of Diastolic Dysfunction and Diastolic Heart Failure [DIAST-CHF]) [2,3], and one was a non-pharmacological randomised controlled trial (the Interdisciplinary Network Heart Failure [INH] programme), which evaluated a remote patient management programme [4,5].

The IKARIUS study was a prospective, non-interventional cohort study recruiting patients with dilative cardiomyopathy (DCM), which was defined as left ventricular ejection fraction (LVEF) <45% and/or left ventricular end-diastolic diameter (LVEDD) >56 mm in the absence of secondary causes of HF [1]. The aim was to investigate the pathogenesis of DCM with respect to expression of causative genes and genetic associations with autoimmune diseases and infections. Exclusion criteria were a lack of written informed consent and inability to participate for logistic reasons.

The DIAST-CHF study was a prospective non-interventional multicentre cohort study investigating the prevalence and clinical course of heart failure (HF) in outpatients with a history of HF, asymptomatic diastolic dysfunction confirmed by echocardiography, or ≥1 cardiovascular risk factor (e.g. hypertension, diabetes mellitus, sleep apnoea syndrome, obesity and/or coronary disease) [2,3]. Exclusion criteria were a lack of written informed consent or inability to participate for logistic reasons.

The INH programme was a randomised, controlled, multicentre trial investigating the efficacy of nurse-coordinated remote patient management (HeartNetCare-HF^TM^) in patients discharged from hospital after acute decompensation for systolic HF [4,5]. To be eligible, patients had to have a LVEF ≤40% documented by echocardiography prior to discharge [4]. Important exclusion criteria were: new-onset structural heart disease (e.g. acute myocardial infarction); lack of written informed consent; and logistic reasons precluding participation in a telephone-based intervention.

**Table S1** Competence Network Heart Failure studies contributing patients to the current analysis

|  | **Study design** | **Recruitment**  **period** | **Follow-up period** | **Total recruited** | | |  | **Current analysis** | | | | |
| --- | --- | --- | --- | --- | --- | --- | --- | --- | --- | --- | --- | --- |
|  |  |  |  | **n** | **Age, years**  **mean (SD)** | **Female,**  **%** |  | **n** | **ACC/AHA stage, n** | | | |
|  |  |  |  |  |  |  |  |  | **A** | **B** | **C1** | **C2/D** |
| DIAST-CHF [2,3] | Prospective cohort | 2004-2007 | 2010-2014 | 1735 | 67 (8) | 48 |  | 1274 | 202 | 881 | 148 | 43 |
| IKARIUS [1] | Prospective cohort | 2004-2008 | 2010-2012 | 322 | 51 (13) | 25 |  | 201 | 0 | 0 | 99 | 102 |
| Interdisciplinary Network Heart Failure study [4,5] | Randomised controlled | 2004-2008 | 2009-2014 | 1022 | 68 (13) | 29 |  | 1021 | 0 | 0 | 575 | 446 |

ACC/AHA = American College of Cardiology/American Heart Association; DIAST-CHF = Diagnostic Trial on Prevalence and Clinical Course of Diastolic Dysfunction and Diastolic Heart Failure; IKARIUS = Inflammatory/Familial Dilated Cardiomyopathy: Is There a Link to Autoimmune Diseases?; SD = standard deviation.

**Table S2** Proportions of patients with chronic kidney disease and/or anaemia in American College of Cardiology/American Heart Association heart failure stages A and B with NT-proBNP levels ≤ 125 pg/ml vs. > 125 pg/ml.

|  | **Stage A** | |  | **Stage B** | |
| --- | --- | --- | --- | --- | --- |
|  | **NT-proBNP** | |  | **NT-proBNP** | |
|  | **≤125 pg/ml** | **>125 pg/ml** |  | **≤125 pg/ml** | **>125 pg/ml** |
|  | N=160 | N=42 |  | N=484 | N=397 |
| **Chronic kidney disease, n (%)** | 29 (18.1) | 16 (38.1) |  | 73 (15.1) | 135 (34.0) |
| **Anaemia, n (%)** | 3 (1.9) | 3 (7.1) |  | 20 (4.1) | 50 (12.6) |

NT-proBNP = N-terminal pro B-type natriuretic peptide.

CKD was defined as eGFR <60 mL/min/1.73m^2^. Anaemia was defined as haemoglobin <12 g/dL (females) or <13 g/dL (males) according to World Health Organization criteria.

**Table S3** NT-proBNP levels in patients with/without loop diuretics across American College of Cardiology/American Heart Association heart failure stages.

|  | **Stage A** | |  | **Stage B** | |  | **Stage C1** | |  | **Stage C2/D** | |
| --- | --- | --- | --- | --- | --- | --- | --- | --- | --- | --- | --- |
|  | **loop diuretics** | |  | **loop diuretics** | |  | **loop diuretics** | |  | **loop diuretics** | |
|  | **no** | **yes** |  | **no** | **yes** |  | **no** | **yes** |  | **no** | **yes** |
|  | **N=191** | **N=11** |  | **N=789** | **N=92** |  | **N=301** | **N=521** |  | **N=119** | **N=472** |
| **NT-proBNP (pg/ml)** | 65 (40; 106) | 43 (29: 182) |  | 104 (53; 192) | 190 (89; 482) |  | 514 (170; 2095) | 1956 (654; 4926) |  | 1648 (466; 4067) | 4894 (1759; 11517) |
| **NT-proBNP > 125 pg/ml, n (%)** | 37 (19.4) | 5 (45.5) |  | 337 (42.7) | 60 (65.2) |  | 201 (82.7) | 387 (94.2) |  | 74 (94.9) | 369 (98.7) |

Values are median (quartiles), or number of patients (%).

NT-proBNP = N-terminal pro B-type natriuretic peptide.

CKD was defined as eGFR <60 mL/min/1.73m^2^. A was defined as haemoglobin <12 g/dL (females) or <13 g/dL (males) according to World Health Organization criteria

**Table S4** Antihypertensive/heart failure medication prescription in subgroups defined by American College of Cardiology/American Heart Association (ACC/AHA) stages and the presence or absence of chronic kidney disease (CKD) and/or anaemia (A).

| **Prescription,  n/n (%)** | **ACC/AHA stage (n)** | | | | | | | | **Global**  **p-value†** |
| --- | --- | --- | --- | --- | --- | --- | --- | --- | --- |
|  | **A (n=201)** | **p-value*** | **B (n=877)** | **p-value*** | **C1 (n=822)** | **p-value*** | **SC2/D (n=591)** | **p-value*** |  |
| **ACEI and/or ARB** | |  |  |  |  |  |  |  |  |
| CKD–/A– | 69/153 (45.1) | 0.037 | 387/632 (61.2) | <0.001 | 427/470 (90.9) | 0.124 | 195/209 (93.3) | <0.001 |  |
| CKD+ and/or A+ | 30/48 (62.5) |  | 182/245 (74.3) |  | 308/352 (87.5) |  | 317/381 (83.2) |  |  |
| **Beta-blocker** |  |  |  |  |  |  |  |  |  |
| CKD–/A– | 48/153 (31.4) |  | 334/635 (52.6) |  | 386/470 (82.1) |  | 167/209 (79.9) |  | 0.292 |
| CKD+ and/or A+ | 15/49 (30.6) |  | 144/246 (58.5) |  | 290/352 (82.4) |  | 305/382 (79.8) |  |  |
| **Mineralocorticoid receptor antagonist** | | |  |  |  |  |  |  |  |
| CKD–/A– | 1/153 (0.7) | 0.418 | 6/653 (0.9) | 0.203 | 177/470 (37.7) | 0.376 | 121/209 (57.9) | <0.001 |  |
| CKD+ and/or A+ | 1/49 (2.0) |  | 5/246 (2.0) |  | 122/352 (34.7) |  | 157/382 (41.1) |  |  |
| **Thiazide diuretic** |  |  |  |  |  |  |  |  |  |
| CKD–/A– | 50/153 (32.7) | 0.011 | 265/635 (41.7) | 0.059 | 132/470 (28.1) | 0.043 | 59/209 (28.2) | 0.011 |  |
| CKD+ and/or A+ | 26/49 (53.1) |  | 120/246 (48.8) |  | 77/352 (21.9) |  | 73/382 (19.1) |  |  |
| **Loop diuretic** |  |  |  |  |  |  |  |  |  |
| CKD–/A– | 8/153 (5.2) |  | 42/635 (6.6) |  | 250/470 (53.2) |  | 135/209 (64.6) |  | <0.001 |
| CKD+ and/or A+ | 3/49 (6.1) |  | 50/246 (20.3) |  | 271/352 (77.0) |  | 337/382 (88.2) |  |  |

*For comparison between CKD–/A– and CKD+ and/or A+ for each medication/medication class in each ACC/AHA stage.

**†**In case of a non-significant interaction between “presence or absence of CKD and/or A” and ACC/AHA stage and a stage constant effect CKD–/A– vs CKD+ and/or A+.

ACEI = angiotensin converting enzyme inhibitor; ARB = angiotensin receptor blocker.

CKD was defined as eGFR <60 mL/min/1.73m^2^. A was defined as haemoglobin <12 g/dL (females) or <13 g/dL (males) according to World Health Organization criteria.

**Table S5** Sensitivity analyses of the impact of chronic kidney disease and anaemia on 5-year survival

|  | **Hazard ratio (95% CI); p-value** |
| --- | --- |
|  | **Adjusted for LVEF and NT-proBNP** |
| CKD+ vs CKD– | 1.8 [1.4-2.1]; p<0.001 |
| A+ vs A– | 1.4 [1.2-1.7]; p=0.001 |
| CKD+/A+ vs CKD–/A–* | 2.5 [2.1-3.0]; p<0.001 |
|  | **Adjusted for LVEF, NT-proBNP, age and sex** |
| CKD+ vs CKD– | 1.4 [1.2-1.8]; p<0.001 |
| A+ vs A– | 1.3 [1.1-1.6]; p=0.008 |
| CKD+/A+ vs CKD–/A–* | 1.9 [1.4-2.5]; p<0.001 |
|  | **Adjusted for LVEF, NT-proBNP, age, sex, leukocytes, diabetes mellitus, malignancy and COPD** |
| CKD+ vs CKD– | 1.4 [1.1-1.7]; p=0.002 |
| A+ vs A– | 1.3 [1.1-1.6]; p=0.012 |
| CKD+/A+ vs CKD–/A–* | 1.8 [1.3-2.3]; p<0.001 |

A = anaemia; CI = confidence interval; CKD = chronic kidney disease; COPD = chronic obstructive pulmonary disease; LVEF = left ventricular ejection fraction; NT-proBNP = N-terminal pro B-type natriuretic peptide.

+ = present; – = absent.

*Linear combination (product) of the effects of CKD and A.

**Table S6** Baseline characteristics of patients who died during follow-up and survivors by American College of Cardiology/American Heart Association stages

|  | Stage A/B (n = 1083) | | Stage C1 (n = 822) | | Stage C2/D (n = 591) | |
| --- | --- | --- | --- | --- | --- | --- |
|  | **Survived**  **(n=1013)** | **Died**  **(n=70)** | **Survived**  **(n=602)** | **Died**  **(n=220)** | **Survived**  **(n=292)** | **Died**  **(n=299)** |
| Demographics/clinical characteristics | |  |  |  |  |  |
| Age years | 66 (60; 71) | 76 (68; 81) | 64 (54; 71) | 73 (64; 78) | 66 (53; 74) | 75 (68; 81) |
| Female, n (%) | 485 (47.9) | 24 (34.3) | 156 (25.9) | 57 (25.9) | 116 (39.7) | 95 (31.8) |
| Systolic blood pressure, mmHg | 150 (137; 163) | 146 (135; 165) | 125 (110; 140) | 120 (110; 140) | 120 (110; 130) | 120 (110; 130) |
| Heart rate, beats/min | 68 (62; 78) | 72 (64; 80) | 72 (63; 80) | 72 (64; 80) | 72 (64; 80) | 72 (64; 80) |
| Body mass index, kg/m^2^ | 29 (26; 32) | 28 (25; 30) | 27 (25; 30) | 26 (24; 29) | 27 (24; 31) | 26 (23; 30) |
| NYHA class |  |  | 2.0 (2.0; 2.0) | 2.0 (2.0; 2.0) | 3.0 (3.0; 3.0) | 3.0 (3.0; 3.0) |
| NYHA class, n (%) |  |  |  |  |  |  |
| I |  |  | 83 (13.8) | 15 (6.8) |  |  |
| II |  |  | 519 (86.2) | 205 (93.2) |  |  |
| III |  |  |  |  | 280 (95.9) | 264 (88.3) |
| IV |  |  |  |  | 12 (4.1) | 35 (11.7) |
| NT-proBNP‡‡, pg/mL | 90 (49; 178) | 209 (103; 480) | 912 (256; 2600) | 3126 (1188; 8134) | 2239 (719; 6352) | 6221 (2617; 15343) |
| NT-proBNP>125 pg/mL, n (%) | 390 (38.5) | 49 (70.0) | 411 (86.9) | 177 (97.8) | 196 (96.1) | 247 (99.6) |
| Comorbidities/risk factors, n (%) |  |  |  |  |  |  |
| Arterial hypertension* | 987 (97.4) | 69 (98.6) | 453 (75.2) | 180 (81.8) | 201 (68.8) | 237 (79.5) |
| Coronary artery disease | 181 (17.9) | 19 (27.1) | 238 (39.5) | 133 (60.5) | 115 (39.4) | 175 (58.5) |
| Obesity† | 381 (37.8) | 19 (27.5) | 172 (28.7) | 46 (21.3) | 90 (31.0) | 69 (23.6) |
| Diabetes mellitus‡ | 247 (24.5) | 27 (38.6) | 148 (24.6) | 78 (35.5) | 94 (32.2) | 134 (45.0) |
| Chronic kidney disease§ | 221 (21.8) | 32 (45.7) | 152 (25.2) | 108 (49.1) | 118 (40.4) | 205 (68.6) |
| Anaemia\|\| | 63 (6.2) | 13 (18.6) | 107 (17.8) | 71 (32.3) | 63 (21.6) | 133 (44.5) |
| Atrial fibrillation | 26 (2.6) | 6 (8.6) | 107 (17.9) | 65 (29.8) | 71 (24.5) | 112 (37.6) |
| COPD^#^ | 58 (5.7) | 6 (8.6) | 68 (11.3) | 38 (17.3) | 36 (12.3) | 81 (27.1) |
| Malignancy** | 76 (7.5) | 14 (20.0) | 56 (9.3) | 30 (13.6) | 25 (8.6) | 49 (16.4) |
| Peripheral arterial disease | 43 (4.2) | 8 (11.4) | 38 (6.3) | 36 (16.4) | 23 (7.9) | 53 (17.7) |
| Echocardiography |  |  |  |  |  |  |
| LVEF, % | 60 (55; 65) | 60 (54; 65) | 35 (27; 40) | 30 (26; 37) | 30 (25; 37) | 30 (23; 35) |
| LVEF <50%, n (%) | 45 (4.4) | 6 (8.6) | 507 (84.2) | 207 (94.1) | 265 (90.8) | 294 (98.3) |
| LV hypertrophy††, n (%) | 754 (75.8) | 46 (70.8) | 457 (82.5) | 171 (87.2) | 230 (86.8) | 238 (91.5) |
| LV dilatation, n (%) | 82 (8.2) | 10 (15.4) | 326 (57.3) | 132 (64.4) | 189 (68.2) | 202 (74.5) |
| Diastolic dysfunction, n (%) | 177 (17.5) | 27 (38.6) | 116 (19.3) | 49 (22.3) | 47 (16.1) | 59 (19.7) |
| Wall motion abnormalities, n (%) | 148 (14.7) | 19 (27.5) | 362 (62.6) | 165 (77.5) | 203 (70.7) | 219 (76.0) |
| Valvular disease ≥II, n (%) | 88 (8.7) | 8 (11.4) | 127 (21.1) | 75 (34.1) | 79 (27.1) | 140 (46.8) |
| Laboratory parameters |  |  |  |  |  |  |
| Haemoglobin, g/dL | 14.1 (13.3; 14.9) | 13.9 (12.9; 14.9) | 14.2 (13.0; 15.2) | 13.5 (12.1; 14.8) | 13.8 (12.7; 15.1) | 12.8 (11.5; 14.2) |
| eGFR, mL/min/1.73m^2^ | 74 (62; 85) | 62 (47; 70) | 73 (60; 90) | 61 (43; 81) | 65 (51; 85) | 49 (34; 66) |
| Leukocytes, 10^9^/L | 6.5 (5.5; 7.5) | 7.3 (6.1; 8.5) | 7.2 (6.1; 8.7) | 7.7 (6.5; 9.9) | 7.9 (6.4; 9.6) | 8.3 (6.7; 10.3) |
| C-reactive protein, mg/L | 1.7 (0.9; 3.5) | 2.1 (1.0; 3.9) | 5.0 (1.9; 11.0) | 7.8 (3.3; 20.2) | 7.0 (4.2; 19.0) | 14.6 (5.5; 37.4) |
| Total cholesterol, mg/dL | 201 (177; 230) | 185 (159; 220) | 185 (155; 213) | 163 (139; 201) | 175 (147; 216) | 160 (128; 188) |
|  |  |  |  |  |  |  |
| Antihypertensive/heart failure medications, n (%) | |  |  |  |  |  |
| ACEI and/or ARB | 627 (61.9) | 41 (58.6) | 545 (90.5) | 190 (86.4) | 266 (91.1) | 246 (82.6) |
| Beta-blocker | 506 (50.0) | 35 (50.0) | 501 (83.2) | 175 (79.5) | 251 (86.0) | 221 (73.9) |
| Mineralocorticoid receptor antagonist | 12 (1.2) | 1 (1.4) | 220 (36.5) | 79 (35.9) | 155 (53.1) | 123 (41.1) |
| Thiazide | 434 (42.8) | 27 (38.6) | 169 (28.1) | 40 (18.2) | 79 (27.1) | 53 (17.7) |
| Loop diuretic | 79 (7.8) | 24 (34.3) | 351 (58.3) | 170 (77.3) | 210 (71.9) | 262 (87.6) |

Values are median (quartiles), or number of patients (%).

ACEI = angiotensin converting enzyme inhibitor; ACC/AHA = American College of Cardiology/American Heart Association; ARB = angiotensin receptor blocker; COPD = chronic obstructive pulmonary disease; eGFR = estimated glomerular filtration rate; LV = left ventricular; LVEF = left ventricular ejection fraction; NT-proBNP = N-terminal pro b-type natriuretic peptide; NYHA, New York Heart Association.

*Arterial hypertension was defined as systolic blood pressure ≥ 140 mmHg, diastolic blood pressure ≥ 90 mmHg and / or documented history of hypertension.

**†**Obesity was defined as body mass index ≥30 kg/m^2^.

‡Diabetes mellitus was defined based on antidiabetic therapy and / or history of diabetes mellitus.

§Chronic kidney disease was defined as eGFR <60 mL/min/1.73m^2^.

||Anaemia was defined as haemoglobin <12 g/dL in women, <13 g/dL in men (World Health Organization criteria).

#COPD was defined based on anti-obstructive therapy and / or history of COPD.

**Malignancy denotes cured and uncured malignant disease.

**††**LV hypertrophy was measured in 2335 patients.

‡‡NT-proBNP was measured in 2189 patients (all other variables were measured in > 95% of the study population).

**Table S7** Cause-specific death during follow-up by American College of Cardiology/American Heart Association heart failure stage

|  | **Stage A/B (n=1083)** | **Stage C1 (n=822)** | **Stage C2/D (n=591)** |
| --- | --- | --- | --- |
| All-cause death, n (% of all patients per group) | 70 (6.5) | 220 (26.8) | 299 (50.6) |
| Cause of death unknown, n (% of diseased patients per group) | 29 (41.4) | 41 (18.6) | 55 (18.4) |
| **Cardiovascular death, n (% of diseased patients per group)** |  |  |  |
| Total | 6 (8.6) | 119 (54.1) | 165 (55.2) |
| Myocardial infarction | 2 (2.9) | 8 (3.6) | 21 (7.0) |
| Arrhythmia | 1 (1.4) | 31 (14.1) | 37 (12.4) |
| Decompensated heart failure | - | 57 (25.9) | 81 (27.1) |
| Other cardiovascular cause or cardiovascular death suspected | 3 (4.3) | 14 (6.4) | 17 (5.7) |
| Stroke | - | 8 (3.6) | 5 (1.7) |
| Pulmonary embolism | - | 1 (0.5) | 4 (1.3) |
| **Non-cardiovascular death, n (% of diseased patients per group)** |  |  |  |
| Total | 35 (50.0) | 60 (27.3) | 79 (26.4) |
| Sepsis | 4 (5.7) | 15 (6.8) | 22 (7.4) |
| Cancer | 18 (25.7) | 18 (8.2) | 23 (7.7) |
| Other | 13 (18.6) | 27 (12.3) | 34 (11.4) |

**References**

1. Pankuweit S, Lüers C, Richter A, Ruppert V, Gelbrich G, Maisch B (2015) Influence of different aetiologies on clinical course and outcome in patients with dilated cardiomyopathy. Eur J Clin Invest 45:906-917.

2. Stahrenberg R, Edelmann F, Mende M, Kockskämper A, Düngen HD, Scherer M, Kochen MM, Binder L, Herrmann-Lingen C, Gelbrich G, Hasenfuß G, Pieske B, Wachter R (2010) Association of glucose metabolism with diastolic function along the diabetic continuum. Diabetologia 53:1331-1340.

3. Trippel TD, Mende M, Düngen HD, Hashemi D, Petutschnigg J, Nolte K, Herrmann-Lingen C, Binder L, Hasenfuss G, Pieske B, Wachter R, Edelmann F (2021) The diagnostic and prognostic value of galectin-3 in patients at risk for heart failure with preserved ejection fraction: results from the DIAST-CHF study. ESC Heart Fail 8:829-841.

4. Angermann CE, Störk S, Gelbrich G, Faller H, Jahns R, Frantz S, Loeffler M, Ertl G (2012) Mode of action and effects of standardized collaborative disease management on mortality and morbidity in patients with systolic heart failure. Circ Heart Fail 5:25-35.

5. Albert J, Lezius S, Störk S, Morbach C, Güder G, Frantz S, Wegscheider K, Ertl G, Angermann CE (2021) Trajectories of Left Ventricular Ejection Fraction After Acute Decompensation for Systolic Heart Failure: Concomitant Echocardiographic and Systemic Changes, Predictors, and Impact on Clinical Outcomes. J Am Heart Assoc 10:e017822.
